# Laparoscopic Cystectomy In-a-Bag of an Intact Cyst: Is It Feasible and Spillage-Free After All?

**DOI:** 10.1155/2016/8640871

**Published:** 2016-03-23

**Authors:** Stelios Detorakis, Dimitrios Vlachos, Stavros Athanasiou, Themistoklis Grigoriadis, Aikaterini Domali, Ioannis Chatzipapas, Emmanuel Stamatakis, Athanasios Mousiolis, Apostolos Patrikios, Aris Antsaklis, Dimitrios Loutradis, Athanasios Protopapas

**Affiliations:** 1st Department of Obstetrics and Gynecology of the University of Athens, Alexandra Hospital, 80 Queen Sophie Avenue and Lourou Street, 11528 Athens, Greece

## Abstract

This prospective study was conducted to assess the feasibility of laparoscopic cystectomy of an intact adnexal cyst performed inside a water proof endoscopic bag, aiming to avoid intraperitoneal spillage in case of cyst rupture. 102 patients were recruited. Two of them were pregnant. In 8 of the patients the lesions were bilateral, adding up to a total of 110 cysts involved in our study. The endoscopic sac did not rupture in any case. Mean diameter of the cysts was 5.7 cm (range: 2.3–10.5 cm). In 75/110 (68.2%) cases, cystectomy was completed without rupture, whereas in the remaining 35/110 (31.8%) cases the cyst ruptured. Minimal small spillage occurred despite every effort only in 8/110 (7.2%) cases with large (>8 cm) cystic teratomas. There were no intraoperative or postoperative complications. We concluded that laparoscopic cystectomy in-a-bag of an intact cyst is feasible and oncologically safe for cystic tumors with a diameter < 8 cm. Manipulation of larger tumors with the adnexa into the sac may be more difficult, and in such cases previous puncture and evacuation of the cyst contents should be considered.

## 1. Introduction

Advances in laparoscopic surgery over the past 3 decades have made the removal of most benign ovarian masses that previously required laparotomy technically possible. Laparoscopic surgery is less invasive, requires shorter hospitalization and recovery times, and is usually favored by young patients due to its better aesthetic results [1, 2]. However its role in the management of adnexal masses has become controversial, in terms of the oncological safety of such a procedure. The main concern is that iatrogenic rupture and spillage of contents of a malignant adnexal mass would upgrade the disease stage, resulting in a need for adjuvant chemotherapy and possibly compromising the overall survival of the patient [3, 4]. These concerns have led to several guidelines and restrictions concerning laparoscopic management of adnexal masses throughout the years, which are not generally adopted and change quite often [5, 6].

Laparoscopic cystectomy in-a-bag is a technique proposed for the management of suspicious adnexal cystic masses and has been described in the early '90s [7, 8]. Nevertheless, its real value in preventing spillage of contents in case of intraoperative rupture of a cyst has not been properly assessed in a prospective manner. Furthermore, under the preoperative term “suspicious adnexal masses” a variety of pathologies will be included with the majority representing benign ovarian swellings [9, 10].

Our study was designed to investigate prospectively the true value of a large waterproof and handle-free endoscopic sac in preventing spillage of contents after rupture of laparoscopically managed cystic adnexal masses. We also attempted to determine the probabilities of rupture for each of the several histologically different cystic masses encountered in a group of young patients, in whom an effort to excise the lesion intact was made. Our purpose was to recognize preoperative and intraoperative risk factors for rupture, and spillage after rupture, and set the limits for attempting excision of an intact cyst versus performing puncture and evacuation of its contents.

## 2. Materials and Methods

Patients with cystic adnexal masses referred for laparoscopic management to the Gynecological Endoscopy Unit of the 1st Department of Obstetrics & Gynecology of the University of Athens, “Alexandra” Hospital, Athens, Greece, from January 2009 to September 2013, were recruited for this prospective cohort study. The study was approved by our institution's scientific committee, and a detailed informed consent was obtained from all patients.

The standard preoperative triage included a complete clinical and gynecological examination and tumor markers (CEA, CA-125, CA19-9, a-fetoprotein, and *β*-hCG), plus a detailed pelvic transvaginal (TVS) and/or transabdominal (TAS) ultrasound scan. During ultrasonographic examination the following characteristics of the adnexal mass were looked for and recorded: size, appearance of fluid content, presence of a solid component, septations, papillary projections into the cyst or surface excrescences, the thickness of the cyst wall, presence of neovascularization, and the cyst's uni- or bilateral localization. An abdominal CT scan or MRI was occasionally performed to assist in the differential diagnosis. Women > 45 years old, those with a preoperative diagnosis of an endometrioma, and those with probable invasive ovarian cancer were excluded from this study. Cases with possible functional cysts were reexamined after 3-4 months and were scheduled for laparoscopy were the lesion persisted.

Ovarian masses included in our sample were allocated into four groups regarding their sonographic characteristics: fluid filled structures, anechoic or of low echogenicity, possible teratomas, paraovarian cysts, and masses that could not be classified as benign or malignant based on their sonographic appearance. Fluid filled ovarian lesions were subgrouped into unilocular and multilocular. Ovarian lesions presenting with mixed echogenicity, consisting of longitudinal white lines or a Rokitansky nodule, and showing acoustic shadows were categorized as teratomas. Unilocular, anechoic cystic masses observed nearby the ovary were allocated in a separate group. Finally, ovarian cystic lesions that could not be preoperatively reported as definitely benign were characterized as suspicious. Presence of papillary projections, meaning inner irregularities of cystic wall > 3 mm, and presence of a solid component were taken into account. Solid parts, in particular, were differentiated from Rokitansky nodules based on the presence or not of acoustic shadows. Because of the expected high prevalence of benign masses in our group of patients, size per se was not considered a single criterion to characterize the mass as suspicious or not, despite the fact that lesion size may affect preoperative diagnosis [11–13].

Laparoscopic surgery was performed using the technique of 4 trocars: one primary trocar was placed through a vertical intraumbilical incision, allowing the use of a 10 mm, 0-degree laparoscope. Another two accessory trocars (5 mm) were inserted in the lower abdomen lateral to the inferior epigastric vessels and a fourth accessory trocar (5 mm) was inserted above the pubic hairline.

**Figure 1:**
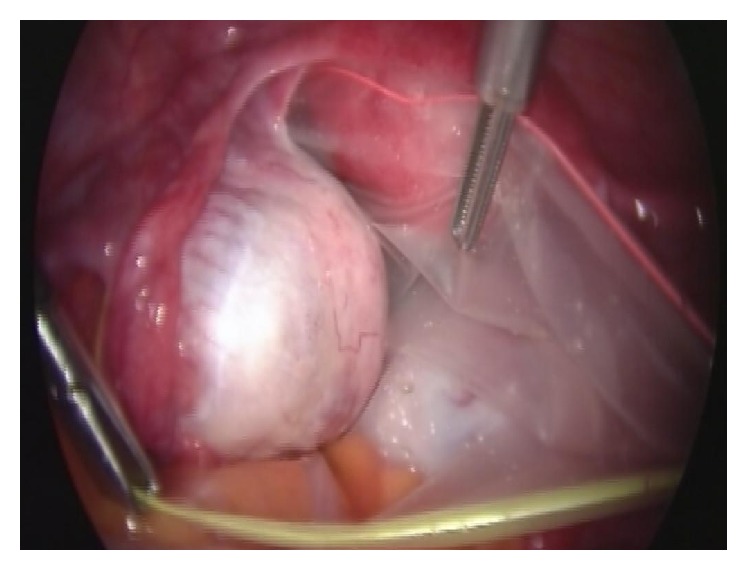
Cyst-harboring adnexa inside handle-free endoscopic sac.

After insertion of the laparoscope, a careful and thorough inspection of the pelvis and abdomen was performed and peritoneal washings or free peritoneal fluid was taken for cytological examination. The waterproof endoscopic bag (Unimax, Medical Technology Promedt, Consulting GmBH, 5′′  ×  7′′) was inserted blindly into the peritoneal cavity wrapped tightly into its plastic applicator through the umbilical trocar and opened by unrolling it with atraumatic forceps, after replacing the laparoscope. The lesion-harboring adnexa was placed and kept inside the bag throughout its dissection (Figure 1). The endoscopic bag used in our study was not attached to an external manipulator; instead it was supplied with a lasso at its rim made of a memory wire aimed to be tightened in the end of the procedure to allow for safe specimen extraction. Laparoscopic cystectomy was performed without previous evacuation of the cyst, making an effort to keep the adnexa inside the sac throughout the procedure and excise the cyst intact. In case of inadvertent rupture even minimal leakage was recorded. In cases with bilateral cysts the side harboring the smaller cyst was treated first.

The ovarian capsule was incised with scissors and the cleavage plane between cyst and ovary was identified. The cyst was enucleated from the surrounding ovarian tissue mainly by means of blunt dissection and/or aqua dissection. Dense adhesions between the cyst and ovarian stroma were divided sharply with scissors after bipolar coagulation, closer to the surface of the cyst. In cases with suspicious masses the technique was slightly modified and the adhesions were divided closer to the ovary than the cyst to allow for a safety margin. In case of rupture during cyst dissection the instrument tips were washed with normal saline, while inside the bag, before removing them from the peritoneal cavity.

After excision of the cyst, the left 5 mm accessory trocar was replaced by a 10 mm trocar. The end of the wire was pulled outside the peritoneal cavity through this trocar which was removed, and the closed mouth of the bag was retrieved through the skin incision and opened extracorporeally. The cyst was deflated inside the bag using a needle or another cutting instrument and a suction pump, in order to reduce its volume and make the extraction of the bag and the remaining cyst possible without contamination of the abdominal wall. Whenever a solid component was prominent the incision was enlarged to allow for easier and safer extraction.

Based on final histology the following histological groups emerged and are used for analysis: serous cystadenoma, mucinous cystadenoma, benign cystadenofibroma, simple serous cyst, benign mature cystic teratoma, paraovarian cyst, and borderline ovarian tumor.

## 3. Statistics

Statistical analysis was performed with the Statistics Package for Social Sciences (SPSS) version 15. An independent samples *t*-test and the nonparametric Mann-Whitney *U* test were used to compare MCDs between cases with and without rupture, and between cases with and without spillage. The Chi-square and Fisher's exact tests were used to determine the statistical significance during comparisons of categorical data.

The logistic regression model provided the estimated probability of rupture and spillage for any particular case. In this model the MCD was used as an independent variable. A *p* value of less than 0.05 was considered as statistically significant.

## 4. Results

All 102 cases included in this study were operated on by a single senior surgeon (AP) to ensure consistency in the operative technique. In 8 cases the lesions were bilateral, which made a total of 110 cysts available for analysis. Two patients were pregnant and their procedure was performed during the second trimester of pregnancy. Another 6 cases, 4 with endometriomas and 2 with functional cysts with an erroneous preoperative diagnosis discovered during surgery, were excluded from this study.

Patients and maximum cyst diameters per group of final histology are summarized in Table 1. Mean patient age was 28.9 years (range: 12–44). Mean maximum diameter of the cysts (MCD) was 5.7 cm (range: 2.3–10.5 cm). The endoscopic sac did not rupture in any case. In 75/110 (68.2%) cases, cystectomy was completed without rupture, whereas in the remaining 35/110 (31.8%) cases the cyst ruptured. Spillage occurred despite every effort in 8/110 (7.2%) cases, all with large (>8 cm) cystic teratomas. Rupture occurred in 32.1% (17/53) of the cysts with sonolucent fluid content, whereas, in 26.4% (14/53) of those with mixed solid and sonolucent contents and in 100% (4/4) of those with internal echos, the cyst ruptured.

**Figure 2:**
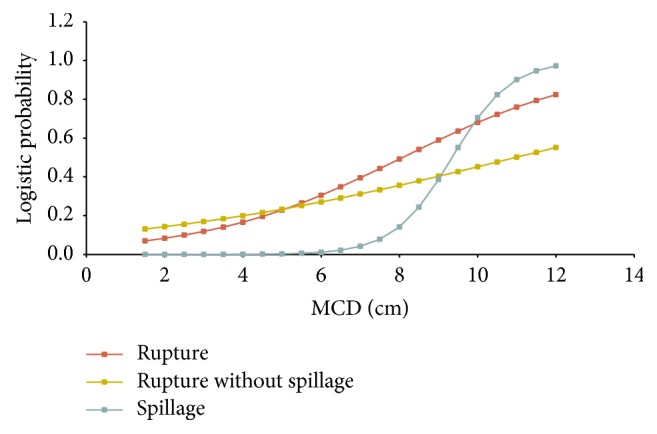
Probabilities of rupture with and without spillage in relation to MCD (cm).

The mean MCD of the cysts that ruptured independent of spillage was 6.75 cm and the mean MCD of those without rupture was 5.60 cm. This difference was statistically significant (*p* < 0.001). In the group of patients with rupture but no spillage the MCD was 6.10 cm and did not differ significantly compared with that of the no-rupture group. When attempting to determine a cutoff point of the MCD above which the probability of rupture (with or without spillage) increases significantly, this was set at 7.3 cm. Among ovarian cysts with a MCD ≥ 7.3 cm 57.7% (15/26) ruptured, compared with 23.8% (20/84) of those with a MCD < 7.3 cm (*p* = 0.003). The relative risk (RR) of rupture was 4.36 times greater for cysts with MCD ≥ 7.3 cm than for those with MCD < 7.3 cm (Figure 2).

**Figure 3:**
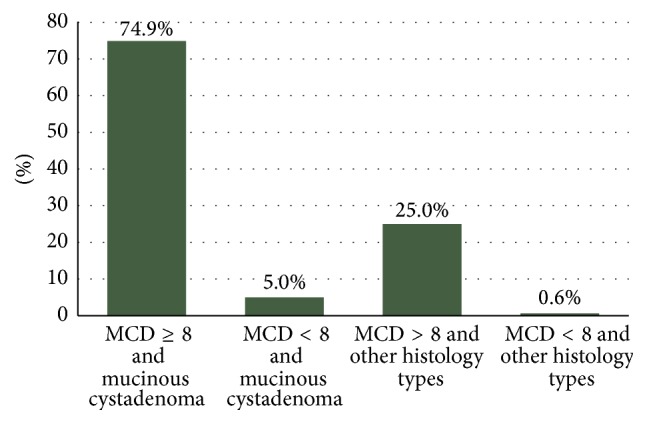
Probabilities of rupture in relation to MCD (cm) and cyst histology.

**Figure 4:**
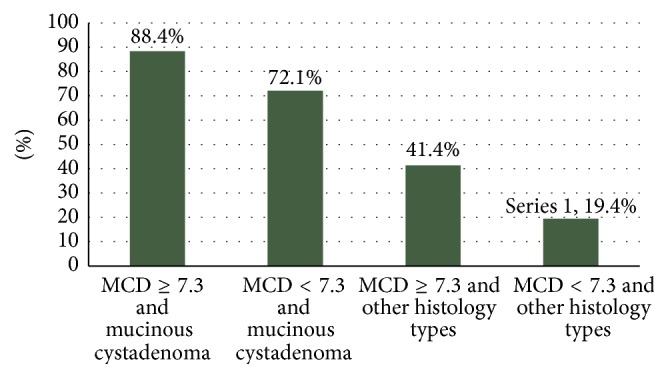
Probabilities of spillage in relation to MCD (cm) and cyst histology.

Combining the final histological diagnosis and the MCD of the cyst with the probability of rupture, we found that ovarian cysts with a MCD ≥ 7.3 cm had an almost threefold (×2.94) higher RR of rupture than those with a MCD < 7.3 cm for a given histological type (*p* = 0.040). Mucinous cystadenomas in particular were more likely to rupture during their excision compared with other histological types of the same diameter; 81.3% (13/16) of the mucinous cystadenomas ruptured, whereas only 23.4% of the other histological types (22/94) did so during their excision (*p* < 0.0001). The RR of rupture for mucinous cystadenomas was 10.7 times higher compared with other histological types, for the same MCD (Figures 3 and 4).

In our study 31.8% of the ovarian cysts (35/110) ruptured during their excision with the previously described technique. In 22.9% (8/35) of these, spillage of their contents into the peritoneal cavity was recorded, and therefore the percentage of rupture and spillage in our study was 7.2% (8/110). The mean MCD of the ovarian cysts with spillage of their contents was 8.94 cm, which was significantly higher than the mean MCD of 5.74 cm of those without spillage (independent of rupture), and the mean MCD of 6.10 cm of those with rupture but no spillage (*p* < 0.0001, each comparison). In the group with rupture and spillage 5 of the 8 (62.5%) cases had mucinous cystadenomas. Moreover, 31.3% (5/16) of mucinous cystadenomas sustained spillage of their content. The percentage of spillage in all other histological types grouped together was significantly lower (*p* = 0.002).

We also tried to determine a cutoff point of statistical significance (according to what we did for cyst rupture) above which the probability of spillage of contents of a ruptured ovarian cyst into the peritoneal cavity increases significantly. This cutoff point was set at 8 cm. Among ovarian cysts with MCD ≥ 8 cm, 43.8% (7/16) sustained spillage, compared with only 1.1% (1/94) of those with MCD < 8 cm (*p* < 0.0001). In other words, the RR of spillage was 72 times higher for cysts ≥ 8 cm than for those < 8 cm. In the group of 35 cysts that ruptured during their excision and in relation to the cutoff point of 8 cm, in only 3.6% (1/28) of those with a MCD < 8 cm their contents were spilled after rupture, compared with 100% (7/7) of those with MCD ≥ 8 cm (*p* < 0.001).

Regarding the relation between the histological diagnosis of the cyst and the probability of spillage, we found that cysts with MCD ≥ 8 cm had a RR for spillage 56 times higher than those with MCD < 8 cm, among cysts with the same final histology (FH) (*p* = 0.001). Mucinous cystadenomas in particular, for the same MCD, had a RR for spillage 8.9 times higher than other histological types (*p* = 0.033). Borderline tumors, in particular, ruptured in 2/4 (50%) cases, including a pregnant patient, but without any spillage, whereas in none of the 11 paraovarian cysts ruptured.

Another interesting aspect in our study was to investigate how the increase of the MCD affects the probability of rupture and spillage. In order to determine that aspect, we developed three statistical models using as an independent variable the MCD and as a dependent variable the event of rupture with or without spillage (model 1), rupture without spillage (model 2), and finally rupture with spillage (model 3). For every 1 cm increase of the MCD, there is an average increase of the RR of rupture by 1.48 times (*p* = 0.02). For every 1 cm increase of the MCD, there is an average increase of the RR of rupture with spillage by 3.8 times (*p* = 0.001) (Figure 2).

## 5. Discussion

This prospective study was conducted to determine safety criteria for attempting the laparoscopic excision of an intact adnexal cyst using a handle-free endoscopic sac as protection from spillage. Our study population consisted exclusively of young patients desiring preservation of their full reproductive capacity. We believe that avoidance of spillage during cystectomy is of great importance not only for malignant cysts (because of disease upstaging), but also for benign ones, because of the possibility that the spilled content might cause chemical peritonitis and result in future periadnexal and intraperitoneal adhesions, even in the absence of symptoms [14, 15].

The operative management of adnexal cystic swellings (ACS) represents one of the commonest indications for laparoscopic gynecological surgery. Such a preoperative diagnosis may include several pathological entities: ovarian and nonovarian lesions, nonneoplastic and neoplastic masses, and, among these, benign, borderline, and even invasive neoplasms. Accurate preoperative and/or intraoperative diagnosis may be feasible on many occasions and impossible in others, for the reason that different pathologies may share a variety of similar morphological features [16–18].

“Suspicious adnexal mass” is a term used to describe a lesion that does not appear to be overt cancer but possesses several sonographic or morphologic characteristics that increase its likelihood of proving malignant at final histology [7, 19]. Despite the fact that even today many authorities consider laparoscopy an inappropriate tool to treat invasive ovarian cancer, the laparoscopic approach has been established over the years as the first-line operative modality to evaluate suspicious adnexal masses [19–21].

The prevalence of invasive ovarian cancer is highly variable in groups of patients with ovarian cystic swellings treated with laparoscopy. It depends on the studied population and is lowest in young patients < 40 years old [19, 22]. In this reproductive age group, laparoscopic cystectomy with ovarian preservation represents the treatment of choice for all benign lesions. After careful patient selection, even suspicious cystic masses may be treated conservatively, providing that all measures are taken to avoid intraoperative spillage in case of rupture. Obviously, removal from the peritoneal cavity of an intact cyst with extraperitoneal evacuation inside a waterproof endoscopic sac has the lowest risk of intraperitoneal spillage and contamination.

The main parameter that should be considered when choosing the right method to excise a cyst (with or without previous evacuation of its contents) is its maximal diameter. Rupture of a cyst does not necessarily lead to spillage of its contents into the peritoneal cavity, providing that it is always being excised in a waterproof laparoscopic sac, whereas rupture is obviously a prerequisite for spillage. Our study showed that the RR for rupture increases by 48% for every 1 cm rise in cyst diameter, whereas the RR for spillage quadruples, respectively. From a clinical perspective, the main concern is not so much to avoid rupture of a cyst but to avoid spillage. Therefore the cutoff point in cyst diameter with a major clinical significance was set at 8 cm. Based on our results, showing that 43.8% of the cysts with MCD ≥ 8 cm sustained spillage of their contents, compared with only 1.1% of those < 8 cm (in other words the RR of spillage was 72 times higher for cysts ≥ 8 cm), we can conclude that the technique of excision of an intact cyst, without previous evacuation of its contents, is effective and oncologically safe for lesions ≤ 8 cm. For larger ovarian cystic masses it is recommended to puncture and evacuate the cyst inside the sac and then remove its residual wall from the ovary.

Mucinous cystadenomas (the majority of which were multilocular) were associated with an almost 10-fold higher RR for rupture compared with other histological types (for the same MCD). It can be safely concluded that for cysts that give us morphologically the impression of a mucinous cystadenoma, it is safer to previously evacuate their content (always in a laparoscopic bag in order to avoid microspillage) and then excise the remaining cystic wall. This may require more than a single puncture.

An interesting finding of our study was the low rate of rupture for those cystic masses that were characterized as suspicious. Overall, only 3/14 (21.4%) cysts in this group (4 borderlines, 5 cystadenofibromas, 2 serous cystadenomas, and 3 teratomas) ruptured, with 0% spillage. For this group of patients the cystectomy technique, as it was mentioned above, was slightly different. With the modified technique, even in the group of patients with borderline ovarian tumors (*N* = 4), the rupture rate was 50% (one in a pregnant patient) with 0% spillage. This indicates (a) that laparoscopic cystectomy may be performed safely in a BOT without spillage and (b) that oophorectomy should not be the obligatory treatment of choice for suspicious cystic masses.

In conclusion, taking into account two major parameters in an adnexal cyst to be treated with laparoscopic cystectomy (maximum diameter and morphological profile) we were able to come up with a guideline concerning choice of the proper technique for its safe excision without spillage of its contents into the peritoneal cavity. Similarly, with proper patient selection, rupture could be avoided in a significant percentage of cases. In any case the adnexa harboring the lesion should be placed inside a waterproof laparoscopic sac and particular attention must be paid to keep it inside the sac throughout the procedure. In the unfortunate event that spillage occurs, cyst contents must be immediately removed by repeated washing and aspiration. Vigorous irrigation of the peritoneal cavity with saline solution, combined with positioning of the patient in the anti-Trendelenburg position by the end of the surgery, minimizes the risk of chemical peritonitis and possibly implantation of malignant cells.

## Figures and Tables

**Table 1:** Distribution of our cases according to the final histological diagnosis.

Final histology	Rupture		Spillage		MCD		Total *N*
	No *N* (%)	Yes *N* (%)	No *N* (%)	Yes *N* (%)	Mean cm	Range cm	
Serous cystadenoma	10 (62.5)	6 (37.5)	14 (87.5)	2 (12.5)	6.98	4.8–9.5	16
Mucinous cystadenoma	3 (18.8)	13 (81.3)	11 (68.8)	5 (31.3)	7.61	4.3–10.5	16
Serous cystadenofibroma	5 (83.3)	1 (16.7)	6 (100.0)	0 (0.0)	6.43	5.0–10.0	6
Simple serous cyst	11 (91.7)	1 (8.3)	12 (100.0)	0 (0.0)	4.47	3.2–6.1	12
Benign cystic teratoma	33 (73.3)	12 (26.7)	44 (97.8)	1 (2.2)	5.17	2.3–8.2	45
Borderline ovarian tumor	2 (50.0)	2 (50.0)	0 (0.0)	0 (0.0)	7.40	5.2–8.6	4
Paraovarian cyst	11 (100.0)	0 (0.0)	11 (100.0)	0 (0.0)	6.25	4.0–9.0	11
Total	75 (68.2)	35 (31.8)	102 (92.7)	8 (7.3)	5.70	2.3–10.5	110
